# The Role of Physical Fitness in the Relationship between Nut Consumption and Body Composition in Young Adults

**DOI:** 10.3390/nu13062126

**Published:** 2021-06-21

**Authors:** Miriam Garrido-Miguel, Vicente Martínez-Vizcaíno, Rubén Fernández-Rodríguez, Isabel Antonia Martínez-Ortega, Luis Enrique Hernández-Castillejo, Bruno Bizzozero-Peroni, Marta Carolina Ruiz-Grao, Arthur Eumann Mesas

**Affiliations:** 1 Health and Social Research Center, Universidad de Castilla-La Mancha, 16071 Cuenca, Spain; miriam.garrido@uclm.es (M.G.-M.); Vicente.Martinez@uclm.es (V.M.-V.); IsabelA.Martinez@uclm.es (I.A.M.-O.); luisenrique_med@hotmail.com (L.E.H.-C.); Bruno.Bizzozero@uclm.es (B.B.-P.); marta.ruiz@uclm.es (M.C.R.-G.); arthur.emesas@uclm.es (A.E.M.); 2 Faculty of Nursing, Universidad de Castilla-La Mancha, 02006 Albacete, Spain; 3 Facultad de Ciencias de la Salud, Universidad Autónoma de Chile, Talca 1101, Chile; 4 Instituto Superior de Educación Física, Universidad de la República, Rivera 40000, Uruguay; 5 Postgraduate Program in Public Health, Universidade Estadual de Londrina, Londrina 86038-350, PR, Brazil

**Keywords:** nuts, adiposity, obesity, fitness, mediation, university students

## Abstract

The main objective of this study was to estimate the association between nut consumption and body composition-related measures and to examine whether this relationship is mediated by cardiorespiratory fitness (CRF) and the muscle strength index (MSI) in young adults. A cross-sectional study involving college students (*n* = 354) aged 18–30 years from a Spanish public university was conducted. Body composition and fitness components were assessed using standard methods. Nut consumption was evaluated using a Food-Frequency Questionnaire. ANCOVA models were used to assess the mean differences in physical fitness and body composition by nut consumption categories. Hayes’s PROCESS macro was applied for mediation and interaction analyses adjusted for the main confounders. Young adults with high nut consumption (≥5 portions of 30 g/week) showed significantly higher values of physical fitness components and fat-free mass and lower values of adiposity-related measures than their peers in the lowest categories of nut consumption (˂1 portion/week) (*p* < 0.05). No significant interaction between CRF and nut consumption on body composition was found. In the mediation analysis, CRF and MSI acted as full mediators of the relationship of nut consumption with fat-free mass and waist circumference/height index. Otherwise, CRF and MSI partially mediated the relationship between nut consumption and body mass index and percent of fat mass. Finally, nut consumption, per se, does not appear to have a significant impact on body composition indicators because these associations have been shown to be partially (for BMI and %BF) or entirely (for ratio WC/height and fat-free mass) explained by CRF and MSI.

## 1. Introduction

Current evidence supports the recommendation of tree nut (i.e., almond, pistachio, walnut) and peanut intake for the prevention and nonpharmacological therapy of chronic conditions such as, dyslipidemia, type II diabetes mellitus, hypertension and cardiovascular mortality [1,2,3]. As nuts provide a substantial amount of energy (calories/g) [4] and have a high nutritional density [5], whether nutrient consumption leads to weight gain and increased body fat is a common concern [6,7]. However, given that nuts have low carbohydrates and are rich in polyunsaturated fatty acids (PUFAs) and fibers, the intake of nuts could raise the subsequent perception of satiety [4,8] and, as a consequence, reduce the total amount of energy intake. Indeed, a recent systematic review of randomized controlled trials (RCTs) noted that the intake of nuts or nut products did not lead to weight gain [9]. Although RCTs provide well-controlled higher-quality data, observational studies provide real-world and more generalizable evidence on the risks and benefits of diet-related exposures [10], such as nut consumption.

The frequency of nut consumption is included as an item of the Mediterranean Diet [11] and has been consistently related to other beneficial lifestyle behaviors such as physical fitness [12]. Thus, it is reasonable to assume that such benefits in body composition are due, at least in part, to a clustering effect of healthy behaviors [12,13]. For this reason, it is essential to clarify whether the stable body weight among frequent nut consumers is due to better cardiorespiratory fitness (CRF) or due to the nut consumption itself. In addition, preliminary findings linked acute almond intake before exercise with enhanced endurance performance [14], which suggests that fitness can act as an intermediate variable between nut consumption and weight control. Hence, a deeper understanding of the potential moderating or mediating effects of CRF on the association between nut consumption and body composition is clearly required.

Although many studies have explored the relationship between nut consumption and body weight, few have included young adults (i.e., from 18 to 35 years) [15]. The promotion of healthy dietary habits in early adulthood, in addition to the encouragement of increasing physical activity and reducing sedentary behavior, is an effective strategy for preventing weight gain [16]. This is particularly relevant for university students, for whom barriers to healthy eating are common, such as time constraints, academic stress, and easy access to junk food [17]. In this context, confirming whether the intake of nuts improves the quality of the diet in young adults without increasing adiposity is important for formulating nutritional recommendations for this age group.

Therefore, the present study aimed to analyze whether the frequency of nut consumption is related with waist circumference (WC), body mass index (BMI), body fat percentage (%BF) and fat-free mass in a sample of young adults. Furthermore, the potential mediating or moderating role of indicators of fitness, such as CRF or muscular strength, in these associations, was also examined.

## 2. Material and Methods

### 2.1. Study Design and Populations

This cross-sectional study was based on sociodemographic data, anthropometric data, lifestyle data and data on markers of physical function obtained from first-year university students from the University of Castilla La Mancha, Spain. A total of 560 university students (aged 18–30 years old) were invited to participate in the present study, and 360 (64.3%) accepted the request. In the present analysis, we used data from a subsample of 354 students in which all dataset variables were evaluated. The participants did not differ in sex, age or parental socioeconomic status from those who refused to participate.

### 2.2. Ethics Approval

All included participants met the following criteria: (i) not having any learning difficulties, and (ii) not having any type of mental or physical disorder. The study protocol was validated and approved by the Clinical Research Ethics Committee of The Virgen de la Luz Hospital in Cuenca (Spain) (REG: 2016jPI1116). All participants were informed about the conditions and characteristics of the present study. Additionally, they were asked to sign a consent form as a condition to participate in the present study. 

### 2.3. Study Variables

Body composition variables. Height was determined twice with the participant upright and barefoot, with the sagittal midline at the midline of the stadiometer, for this determination we used a Seca Model 222 stadiometer. Weight was also measured twice with the student barefoot and wearing light clothing using a Seca Model 770 scale. BMI was assessed as weight (kg) divided by the square of height (m) (kg/m^2^). WC was evaluated as the mean of 3 determinations made at the midpoint between the last rib and the iliac crest at the end of a normal expiration using a flexible tape; to control for height, the WC/height ratio was used for the analyses [18]. BF% and fat-free mass were measured under monitored humidity conditions and temperature using an 8-electrode Tanita Segmental-418 bioimpedance analysis system (Tanita Corp., Tokyo, Japan). In addition, the determinations were completed after urination, before breakfast, and after a 15 min relaxing period. Data collection was performed by trained nurses to reduce interobserver variability [19].

Physical fitness components were assessed after a 4-min warm-up [20] The Course Navette test (20-m shuttle run test) was completed to assess CRF. Participants had to run between two lines separated by 20 m following the rhythm formed by beeps emitted with a prerecorded audio track. Students started running at a speed of 8.5 km/h, and this pace was increased by 0.5 km/h every minute. The test for each participant was finished when the student stopped due to fatigue or when the participant did not reach the line within the beep time on two successive occasions. Additionally, the maximal oxygen consumption was assessed using the Leger 20-m shuttle-run formula (VO_2_ max) (31.025 + (3.238 × velocity) − (3.248 × age) + (0.1536 × age × velocity)) [21].

Muscle strength index (MSI): A TKK 5401 GripD digital adaptive grip dynamometer (Takeya, Tokyo, Japan) was used to determine handgrip strength in kilograms. The exam was completed twice with the right hand and twice with the left hand; the mean average of the 4 measurements was used for the present study. The standing long jump was used to measure lower body strength. Students stood behind a line with their feet approximately shoulder width apart and jumped as far as possible with both feet. The test measures the distance in centimeters from the starting line to the back of the participant’s heels. In this sense, the best results of 3 repetitions was recorded. Last, with the data of these two exams, to estimate a single indicator of strength corrected by weight, a MSI was determined as the sum of the standardized z-score of handgrip/weight and z-score of the standing long jump.

Nut consumption: The Food-Frequency Questionnaire (FFQ) was utilized to estimate the total intake of nuts [22]. This questionnaire includes 137 items with 9 intake frequencies: never or almost never, 1–3 times per month, once per week, 2–4 times per week, 5–6 times per week, once per day, 2–3 times per day, 4–6 times per day, and more than 6 times per day. Total intake of nuts and total energy intake were calculated using Spanish food composition tables [23].

### 2.4. Statistical Analysis

Student’s *t*-test (continuous variables) and chi-squared test (categorical variables) were used to examine the characteristics of the study sample by sex. To assess the normal distribution of continuous variables, both graphical (normal probability plots) and statistical (Kolmogorov–Smirnov test) processes were used for each continuous variable. All continuous variables fit adequately to a normal distribution.

Initially, bivariate correlation coefficients were used to examine the associations between nut consumption, body composition, CRF and physical fitness components. Subsequently, ANCOVA models were used to test the mean differences in body composition physical fitness components as dependent variables by nut consumption categories (˂1/week, 1–4/week, ≥5/week), controlling for sex and age (model 1), total energy intake (model 2), CRF (VO_2_ max estimate) (model 3) and handgrip strength (model 4). Post hoc multiple comparisons were examined using the Bonferroni post hoc test.

Lastly, after ruling out the interaction effect of nut consumption and physical fitness markers on body composition measures, simple mediation models were conducted. For these statistical analyses, we used PROCESS Macro for SPSS version 3.4 (https://www.processmacro.org/index.html, accessed on 10 February 2021) selecting Model 4 and selecting 5000 bias-corrected bootstrap samples. In these analyses, the first equation (path a) corresponded to the regression coefficients of the mediating variables (CRF and MSI) on the independent variable (nut consumption); the second equation (path c, total effect) was the regression coefficient of the outcome (body composition indicators) on the independent variable. Path b represents the regression coefficient of the outcome on the mediating variable. The relationship of the independent variable with the outcome and the mediator simultaneously was estimated in the third equation (path c’, direct effect). To test the statistical significance of the mediation effect in the parametric approach, we used the Sobel test and the indirect effect (path a * path b). These analyses were adjusted for sex and age.

Analyses were performed using the statistical software package IBM SPSS Statistics 24.0 (SPSS Inc., Chicago, IL, USA), and the statistical significance was set at two-tailed *p* < 0.05.

## 3. Results

Descriptive characteristics (mean ± standard deviation or %) of the study sample by sex are shown in Table 1. All variables varied significantly by sex, except age, BMI, weight status, energy intake and nut consumption categories.

Table 2 shows the Pearson correlation coefficients between nut consumption and body composition variables, physical fitness components and total energy intake. Nut intake was negatively associated with BMI, ratio WC/height and %BF, and positively associated with fat-free mass, physical fitness components (CRF, handgrip strength, standing long jump, MSI) and total energy intake (due to the high nutritional density) [5]. Additionally, physical fitness components were positively associated with fat-free mass, nut consumption and total energy intake and negatively associated with body composition variables (BMI, WC/height and %BF).

**Figure 1:**
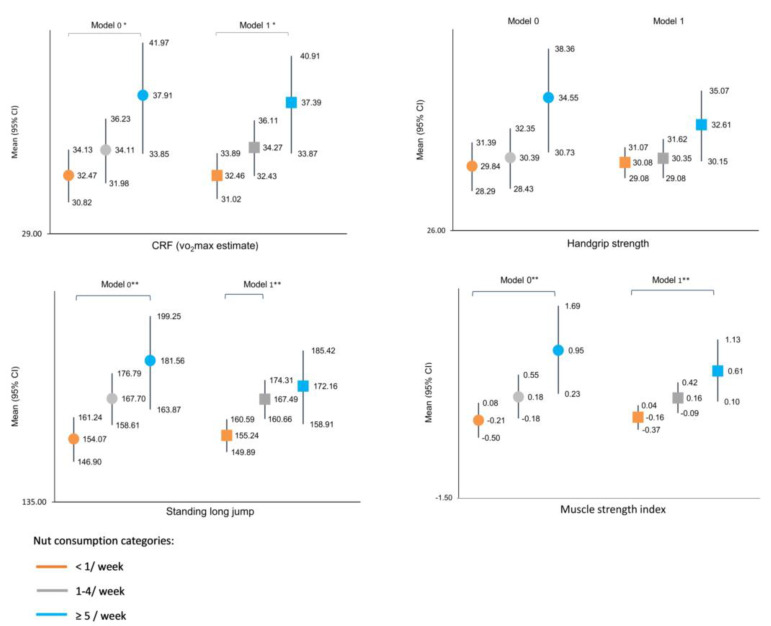
Mean difference and 95% confidence intervals in physical fitness parameters (CRF, handgrip strength, standing long jump and muscle strength index) by categories of nut consumption. O: Model 0: Crude data; □: Model 1: Adjusted by age and sex. * *p* < 0.05, ** *p* < 0.001.

Figure 1 depicts the crude mean differences in the physical fitness components by nut consumption categories (model 0) and after adjusting for sex and age (Model 1). Those with high nut consumption (≥5/week) showed significantly higher values in CRF, handgrip strength, standing long jump and MSI than their peers belonging to the lowest category of nut consumption (˂1/week).

**Figure 2:**
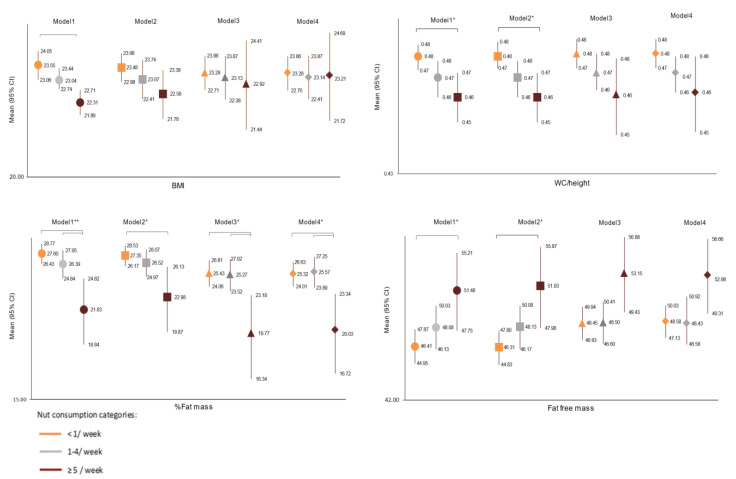
Mean difference and 95% confidence intervals in body composition variables (BMI, WC/height, %fat mass and fat free mass) by categories of nut consumption. O: Model 1: Adjusted by age and sex; □: Model 2: Model 1+ total energy intake; ∆: Model 3: Model 2 + CRF (vo2 max); ◊: Model 4: Model 3 + muscle strength index. * *p* < 0.05, ** *p* < 0.001.

Similarly, Figure 2 shows the adjusted mean differences in the body composition variables by nut consumption categories after controlling for hypothetical confounders (Models 1–4). Those categorized as high nut consumption (≥5/week) showed significantly lower values in WC/height and %BF and higher values in fat-free mass than those categorized as low nut consumption (˂1/week). When we adjusted for sex, age and total energy intake, the differences remained significant (Model 1–2). However, when we adjusted for CRF and MSI (Models 3–4), these differences disappeared for WC/height and fat-free mass variables, suggesting a potential mediating effect of physical fitness in its relationship with nut consumption and these body composition variables.

### 3.1. Interaction Analysis

Figure S1 (Supplemental Material) shows the results from the interaction analysis in which no significant nut consumption × CRF (VO_2_ max) interaction effect on students’ %BF and fat-free mass was observed.

### 3.2. Mediation Analysis

**Figure 3:**
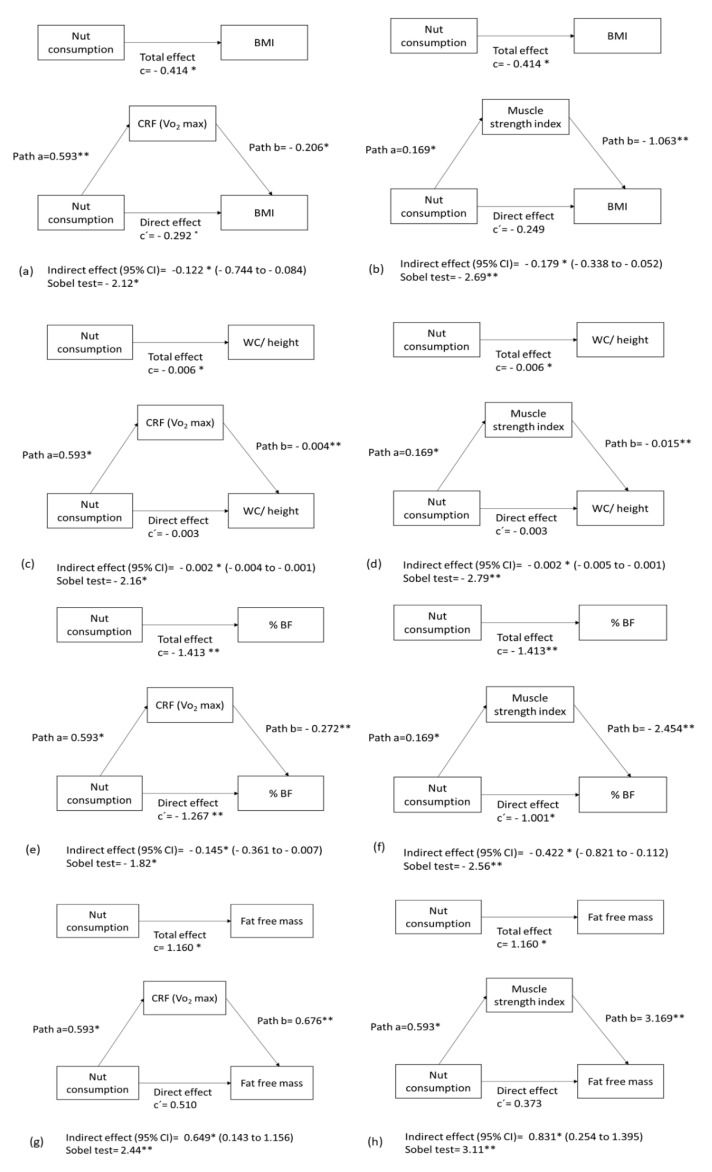
CRF (VO2 max estimate)/muscle strength index mediation models of the relationship between nut consumption with (**a**,**b**) BMI, (**c**,**d**) WC/heigh, (**e**,**f**) %BF, and (**g**,**h**) fat free mass controlling for age and sex. * *p* < 0.05, ** *p* < 0.001.

We assessed the mediating role of CRF (VO_2_ max) and MSI in the relationship between nut consumption and body composition-related measures (Figure 3). In the first regression equation of the mediation model (Figure 3a), nut consumption was positively correlated with CRF (VO_2_ max) (path a = 0.593, *p* < 0.05). In the second regression equation, nut consumption was negatively associated with BMI (path c = −0.414; *p* < 0.05). Finally, in the third regression equation, the relationship between nut consumption and BMI was reduced when the mediator (CRF) was added in the regression model (path c’ = −0.292; *p* ≤ 0.05). Therefore, these findings indicate that the association between nut consumption and BMI was partially mediated by CRF, as confirmed by the values obtained with the Sobel test (z = −2.12, *p* ≤ 0.05). Similar findings were observed when we examined the role of CRF and MSI in the relationship between nut consumption and %BF (Figure 3e,f).

When we examined the mediating role of MSI in the association between nut consumption and BMI (Figure 3b), the results were parallel to those previously described. However, in this case the relationship between nut consumption and BMI was entirely mediated by MSI (Sobel test −2.69, *p* < 0.001). Similar findings were described when we analyzed the role of CRF and MSI in the association of nut consumption with the ratio WC/height (Figure 3c,d) and fat-free mass (Figure 3g,h).

## 4. Discussion

This study examined the role of physical fitness components in the association between nut consumption and body composition-related measures in young adults. Our data preliminary support that (i) high nut consumption (≥5 serving portions/week) is associated with an improvement in physical fitness components compared with low consumption (<1 portion/week); (ii) students with high nut consumption had a lower BMI, ratio WC/height, %BF and higher levels of fat-free mass; however, the association of nut consumption with the ratio WC/height and fat-free mass did not remain after controlling for relevant confounders, including CRF and MSI; and (iii) the benefits of nut consumption on body composition-related indicators are mediated by CRF and MSI.

### 4.1. Nut Consumption and Body Composition-Related Measures

The prospective relationship between nut consumption and lower weight gain in the general population has been previously reported [6,15,16]. Numerous mechanisms have been described by which the intake of an energy-dense food does not lead to an increase in body weight despite the increase in lean mass because nuts also have a high protein content [24]. Among them, the following are worth mentioning [22]: nuts are rich in fiber, which increases satiety and may reduce total energy intake; they are also rich in unsaturated fats, which may increase oxidation and possibly decrease body fat accumulation; moreover, nuts are a source of plant-based proteins, which can positively influence satiety and weight control as well as increase muscle mass and strength [25]; last, not all calories in nuts are used, but a non-negligible proportion is wasted as a consequence of incomplete chewing and digestion [26]. In addition, individuals who regularly consume nuts tend to have healthier overall lifestyles, including better diet quality [27]. For instance, nut consumers have been associated with lower intake of red meats and refined-grain products [6,28].

Thus, our results are consistent with previous evidence suggesting that nut consumption does not increase adiposity. In addition, our results demonstrate that nut consumption may reduce adiposity and increase fat-free mass through improvements in CRF and MSI. To our knowledge, this study is the first to suggest this pathway to understand how the consumption of nuts could benefit body composition.

### 4.2. Nut Consumption and Physical Fitness Components

The effects of nuts on physical fitness have not been completely elucidated, and evidence from well-designed clinical studies is scarce [29]. However, a recent study showed that if exercise is adequately combined with nut supplementation, muscle mass and strength will increase [30], and CRF will improve more effectively [29]. Additionally, the intake of omega-3 PUFAs was suggested to have a positive association with muscle fitness [31] and a beneficial effect on the exercise performance of athletes by reducing the immunomodulatory and inflammatory response to exercise [29,32]. In this sense, Aguilaniu et al. [33] suggested that PUFA supplementation ameliorated exercise-induced hypoxemia. These findings all suggest that unsaturated fatty acids, which are abundant in nuts, have a positive influence on the improvement of physical fitness [33]. In line with this evidence, our study showed a positive association between nut consumption and physical fitness, such that nut consumption of 5 more servings/week was associated with an improvement in the physical fitness components.

### 4.3. Physical Fitness as a Mediator between Nut Consumption and Body Composition-Related Mesasures

The consumption of nuts has been consistently related to other beneficial lifestyle behaviors, such as physical fitness [12], but so far no study has attempted to unravel whether, because fitness is associated with both the intake of nuts and adiposity indicators, it could be considered a confounder, an effect modifier or a mediator. Our mediation analyses, controlling for age and sex, reveal that CRF and MSI act as mediators in the relationship between nut consumption and body composition. Thus, these findings provide preliminary evidence for the hypothesis that physical fitness plays an important role in the relationship between nut consumption and improvements in body composition. Hence, our study reveals that, through its effect on CRF and MSI, nut intake may foster the potential effects of nut consumption on body composition-related measures.

### 4.4. Limitations

Our study has some limitations that are important to highlight. First, the analyses were cross-sectional; hence, we cannot make cause-effect inferences. Second, our sample included only college students (18–30 years-old), so caution is necessary when making inferences to the general or other populations. Third, the use of self-reported dietary information could suffer from underreporting or involuntary measurement errors. Fourth, self-reported consumption of nut intake through the FFQ-137 items could be biased because the items used for estimating the participants’ intake did not differentiate between type of nuts (i.e., almonds, walnuts, pistachios), timing (e.g., at breakfast, mid-morning, lunch) or mode of presentation (e.g., raw, fried, salted); thus, we could not provide more precise evidence regarding the influence of each of these features of nuts on body composition-related measurements. Moreover, although the FFQ-137 items have been validated in the elderly population, further validation questionnaire may be interesting used. Fifth, we were unable to adjust the analyses for other relevant potential confounders, such as the frequency of physical activity, because the models did not fit mainly due to the small number of individuals in the highest category of nut consumption (≥5 servings/week). Finally, further research with other population-based samples, other variables not determined in this study (e.g., sleep quality and social support), and a longitudinal design would help to reveal this association.

## 5. Conclusions

Our data are important from a clinical perspective because they preliminary demonstrate that physical fitness plays a crucial role in the relationship between nut consumption and body composition-related measurements. Nut consumption, per se, does not appear to have a large effect on body composition indicators because these associations have been shown to be partially (for BMI and %BF) or entirely (for ratio WC/height and fat-free mass) mediated by CRF and MSI. This is important from a public health standpoint because they indicate that nut consumption (≥5 servings/week) could result in improvements in body composition-related measures and physical fitness indicators, especially if the intake of nuts is associated with exercise interventions. However, prospective studies are required to confirm the role of nut intake in body composition and whether physical activity interventions using nuts as a source of calories and proteins multiply the improvements in physical fitness.

## Figures and Tables

**Table 1:** Descriptive characteristics of the study sample by sex.

Variable	All (*n* = 354)	Men (*n* = 125)	Women (*n* = 229)	*p* *
Age (years)	21.05 ± 3.11	21.19 ± 2.85	20.97 ± 3.25	0.534
Weight (Kg)	65.41 ± 12.34	72.60 ± 10.94	61.50 ± 11.27	**<0.001**
Height (cm)	167.30 ± 8.66	175.39 ± 7.03	162.93 ± 5.86	**<0.001**
WC (cm)	78.96 ± 9.37	83.13 ± 7.92	76.68 ± 9.40	**<0.001**
Ratio WC (cm)/height (cm)	0.47 ± 0.05	0.47 ± 0.04	0.47± 0.05	**0.002**
% Fat mass	26.61 ± 10.01	18.85 ± 6.83	30.68 ± 8.95	**<0.001**
Fat free mass (Kg)	47.59 ± 10.20	57.93 ± 7.84	42.17 ± 6.41	**<0.001**
BMI (Kg/m^2^)	23.28 ± 3.62	23.55 ± 3.03	23.14 ± 3.90	0.308
Underweight (%)	3.1	0.8	4.4	
Normal weight (%)	70.6	70.6	70.6	0.068
Overweight (%)	21.8	26.2	19.3	
Obesity (%)	4.5	2.4	5.7	
CRF (stages)	5.63 ± 2.67	7.95 ± 2.23	4.09 ± 1.64	**<0.001**
CRF (VO_2_ max estimate, mL/Kg/min)	37.49 ± 8.03	44.44 ± 6.69	32.88 ± 4.94	**<0.001**
Handgrip strength (Kg)	30.41 ± 9.51	39.22 ± 7.75	24.43 ± 4.77	**<0.001**
Standing long jump (cm)	161.21 ± 43.78	195.49 ± 31.91	136.82 ± 33.55	**<0.001**
Muscle strength index (cm/Kg) ^a^	0.013 ±1.7	1.52 ±1.25	−1.05 ± 1.21	**<0.001**
EI (Kcal)	2795.79 ± 1804.77	2865.92 ± 1287.02	2757.68 ± 2033.24	0.590
Carbohydrate (% EI)	43.01 ± 7.10	43.10 ± 6.63	42.95 ± 7.36	0.852
Protein (% EI)	17.47 ± 3.46	17.39 ± 3.23	17.51 ± 3.59	0.749
Fat (% EI)	38.18 ± 6.21	37.93 ± 5.98	38.32 ± 6.34	0.578
Nuts consumption (30 g)	1.59 ± 1.37	1.79 ± 1.47	1.48 ± 1.30	**0.038**
˂1/week	58.8	54.4	61.1	
1–4/week	32.5	32.8	32.3	0.120
≥5/week	8.8	12.8	6.6	

The results are presented as the mean and (±) standard deviation. Bold values suggest statistical significance with *p* < 0.05. Abbreviations: BMI, body mass index; CRF, cardiorespiratory fitness; EI, energy intake; WC, waist circumference; ^a^ Sum of the standardized z score of dynamometry/weight and standing long jump. * Student’s *t*-tests (for continuous variables) and chi-squared tests (for categorical variables).

**Table 2:** Bivariate correlations between nut consumption with body composition, cardiorespiratory fitness (CRF), muscular strength (standing long jump, handgrip strength and muscular strength index) and total energy intake (EI).

	Nut Consumption	BMI	WC/Height	%BF	Fat-Free Mass	CRF (Stages)	CRF (VO_2_ Max Estimate)	Handgrip Strength	Standing Long Jump	Muscle Strength Index ^a^	Total EI
Nut consumption	-	−0.104 *	−0.107 *	−0.216 **	0.151 **	0.178 **	0.177 *	0.129 *	0.206 **	0.199 **	0.396 **
BMI		-	0.845 **	0.493 **	0.340 **	−0.188 **	−0.190 **	0.224 **	−0.066	−0.192 **	−0.116 *
WC/height			-	0.433 *	0.223 *	−0.278 **	−0.278 **	0.081	−0.160 **	−0.232 **	−0.067
%BF				-	−0.537 **	−0.548 **	−0.540 **	−0.390 **	−0.531 **	−0.632 **	−0.166 **
Fat-free mass					-	0.562 **	0.554 **	0.774 **	0.593 **	0.560 **	0.029
CRF (stages)						-	0.996 **	0.597 **	0.705 **	0.739 **	0.188 **
CRF (VO_2_ max estimate)							-	0.589 **	0.697 **	0.732 **	0.186 **
Handgrip strength								-	0.611 **	0.803 **	0.101
Standing long jump									-	0.880 **	0.206 **
Muscle strength index ^a^										-	0.243 **

Data are presented in the correlation coefficient R. * *p* < 0.05, ** *p* < 0.001. Abbreviations: BMI, body mass index; %BF, percentage of body fat mass; EI, energy intake; WC, waist circumference. ^a^ Sum of the standardized z-score of dynamometry/weight and standing long jump.

## Data Availability

The datasets used and/or analyses during the current study are available from the corresponding author (R.F.-R.) on reasonable request.

## Supplementary Materials

The following are available online at https://www.mdpi.com/article/10.3390/nu13062126/s1, Figure S1. CRF (VO2 max estimate) interactions models of the relationship between nut consumption and %BF (a) and fat-free mass (b). Adjusted by age and sex. Beta expressed as unstandardized regression coefficients and 95% confidence interval.
