# Supplementary material for: The Role of Physical Fitness in the Relationship between Nut Consumption and Body Composition in Young Adults
Source: Nutrients. 2021 Jun 21;13(6):2126. doi: 10.3390/nu13062126 (PMC8234532; doi:10.3390/nu13062126)
Supplement: Supplementary file 1 [file nutrients-13-02126-s001.zip › nutrients-1246066-supplementary.pdf]

SUPPLEMENTARY MATERIAL

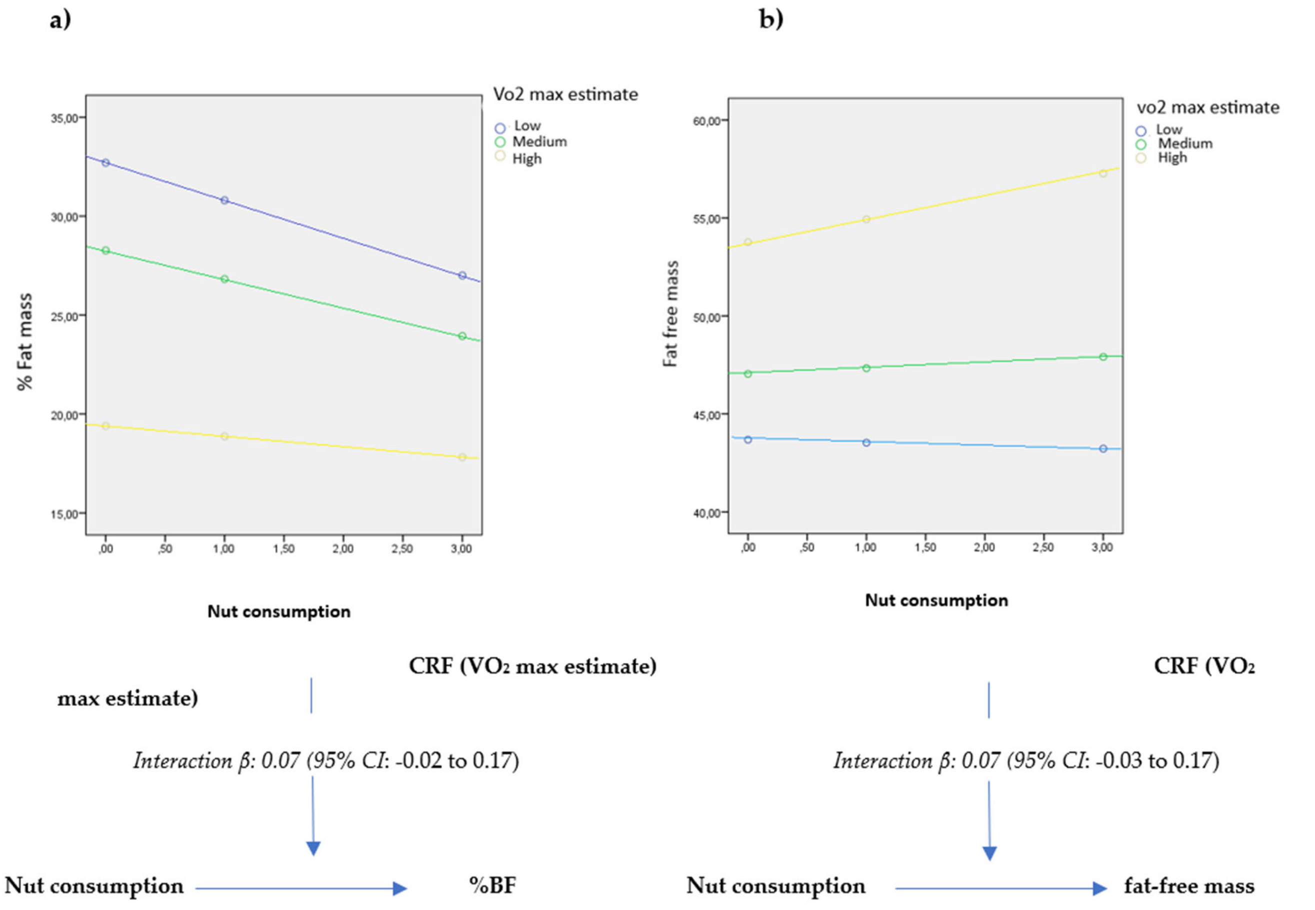

Figure S1. CRF (VO_2 max estimate) interactions models of the relationship between nut consumption and %BF (**a**) and fat-free mass (**b**). Adjusted by age and sex. *Beta* expressed as unstandardized regression coefficients and 95% confidence interval.
